# Artificially sweetened beverage consumption and all-cause and cause-specific mortality: an updated systematic review and dose-response meta-analysis of prospective cohort studies

**DOI:** 10.1186/s12937-024-00985-7

**Published:** 2024-07-31

**Authors:** Zhangling Chen, Cheng Wei, Sander Lamballais, Kang Wang, Yuchan Mou, Yichao Xiao, Fei Luo, Wichor M. Bramer, Trudy Voortman, Shenghua Zhou

**Affiliations:** 1 Department of Cardiovascular Medicine, The Second Xiangya Hospital, Central South University, Changsha, Hunan China; 2 Hunan Key Laboratory of Cardiometabolic Medicine, Changsha, Hunan China; 3 FuRong Laboratory, Changsha, Hunan China; 4 Department of Epidemiology, Erasmus MC, University Medical Center Rotterdam, Rotterdam, the Netherlands; 5 Department of Clinical Genetics, Erasmus MC, University Medical Center Rotterdam, Rotterdam, the Netherlands; 6 Medical Library, Erasmus MC, University Medical Center Rotterdam, Rotterdam, the Netherlands

**Keywords:** Artificially sweetened beverage, Mortality, Dose-response meta-analysis, Prospective cohort studies

## Abstract

**Background:**

Artificially sweetened beverages (ASB) are consumed globally, but their impact on overall health remains uncertain. We summarized published associations between ASB intake with all-cause and cause-specific mortality.

**Methods:**

We searched Medline, Embase, Web of Science, and Cochrane CENTRAL databases until August 2023. Random effect meta-analysis was conducted to calculate pooled risk ratios (RRs) and 95% confidence intervals (95%CIs) for highest versus lowest categories of ASB consumption in relation to all-cause and cause-specific mortality. Linear and non-linear dose-response analyses were also performed.

**Results:**

Our systematic review and meta-analysis included 11 prospective cohort studies. During a median/mean follow-up period of 7.0 to 28.9 years, 235,609 deaths occurred among 2,196,503 participants. Intake of ASB was associated with higher risk of all-cause and CVD mortality with pooled RRs (95%CIs) of highest vs. lowest intake categories of 1.13 (1.06, 1.21) (I^2^ = 66.3%) for all-cause mortality and 1.26 (1.10, 1.44) (I^2^ = 52.0%) for CVD mortality. Dose-response analysis revealed a non-linear association of ASB with all-cause mortality (p_*non−linearity*_ = 0.01), but a linear positive association with CVD mortality (p_*non−linearity*_ = 0.54). No significant association was observed for ASB intake and cancer mortality. Moreover, a secondary meta-analysis demonstrated that replacing 1 serving/day of sugary sweetened beverages (SSB) with ASB was associated with 4–6% lower risk of all-cause and CVD mortality. Per NutriGrade, the evidence quality for associations between ASB intake with all-cause and CVD mortality was moderate.

**Conclusions:**

Higher intake of ASB was associated with higher risk of all-cause and CVD mortality, albeit a lower risk than for SSB.

**Systematic review registration:**

PROSPERO registration no. CRD42022365701.

**Supplementary Information:**

The online version contains supplementary material available at 10.1186/s12937-024-00985-7.

## Introduction

Given the deleterious effects of excess added sugar intake on various health outcomes [1], the World Health Organization (WHO) recommends limiting free sugar consumption to less than 5% of daily energy intake [2]. As a result, artificial sweeteners, characterized by their sweet taste and negligible calorie content, have emerged as alternatives to added sugar and have been widely added to products and accepted by consumers [3]. Given that over 23,000 worldwide products contain artificial sweeteners and that their consumption is high and increasing, the potential health impact of these sweeteners has become an important but controversial topic that has attracted scrutiny from health authorities like the European Food Safety Authority and WHO [4]. Artificially sweetened beverages (ASB), which are among the primary foods containing artificial sweeteners, are often suggested as alternatives to sugary sweetened beverages (SSB). However, the long-term effects of ASB on health remain unclear.

Recently, some randomized controlled trials (RCTs) reported that low-calorie sweeteners facilitated weight loss [5–7]. However, prospective cohort studies have demonstrated higher ASB intakes in relation to higher risks of cardiometabolic diseases, such as obesity, type 2 diabetes, and cardiovascular disease (CVD) [8–10]. Furthermore, several cohort studies have reported harmful or null associations of ASB intake with mortality among the general population [11–17]. These associations have been meta-analyzed, and unfavorable pooled associations were observed between ASB intake with all-cause and CVD mortality among a total of approximately 940,000 participants [18–20]. However, a few recently published cohort studies among large populations have reported null associations [21–24]. For example, Liu et al. [21], Zhang et al. [22], McCullough et al. [23], and Naomi et al. [24] observed null associations between ASB with all-cause, CVD, or cancer mortality among 171,616 participants of the UK Biobank, 31,402 participants of the National Health and Nutrition Examination Survey, 934,777 participants of the Cancer Prevention Study-II (CPS-II) prospective cohort, and 118,707 participants of the Lifelines Cohort Study, respectively. These studies have not been included in the previous meta-analyses, while their combined total of 1,256,502 participants is larger than the overall numbers of participants included in the earlier meta-analyses [18]. In addition, these previous meta-analyses did not further summarize the associations based on the comparison of ASB with other beverages, such as SSB, which may cause to simply imply that ASB is as unhealthy as SSB. Comparing ASB and SSB is crucial to understand the potential health impact of ASB, especially since ASB are often recommended as alternatives to SSB.

Given these additional and inconsistent findings and knowledge gaps, there is a clear need for an updated meta-analysis on ASB consumption and mortality that encompasses both the earlier and newer studies. Such an analysis, coupled with an assessment of the quality of the meta-evidence, could significantly inform the debate on public health measures targeting ASB and artificial sweeteners.

In an effort to comprehensively quantify the associations between ASB intake with all-cause and cause-specific mortality, we performed a systematic review and a dose-response meta-analysis of prospective cohort studies. In addition, we assessed the quality of this meta-evidence using the NutriGrade scoring system [25].

## Methods

### Data sources and searches

We conducted our current study according to Preferred Reporting Items for Systematic Reviews and Meta-Analyses [26], the protocol for this systematic review and meta-analysis was registered on PROSPERO (CRD42022365701). We searched 4 databases: Medline, Embase, Web of Science, and Cochrane CENTRAL through August 2023 (Supplementary Table 1). Also, the references list of selected studies was reviewed to identify additional relevant studies. A study was included for the analysis if it (1) was a prospective cohort study; 2) had assessment of the association between ASB consumption and mortality among generally healthy adults; and 3) provided risk estimates for three or more levels of ASB consumption with mortality or a dose-response estimate. We extracted information on these selected studies, including the first author’s name, publication year, cohort name, study location, follow-up duration, number of participants, sex distribution, age range at baseline, assessment of ASB intake, assessment of outcomes, and covariates. Additionally, the number of deaths, categories of ASB consumption, risk estimates and 95% CIs were derived from the maximally adjusted model. Two investigators (CW and KW) performed double-blind independent screenings of the literature, including title, abstract, and full-text article to identify eligible studies. Disagreement and discordance were discussed until a consensus was reached.

### Statistical analysis

We used the Newcastle-Ottawa scale for cohort studies to assess the quality of the selected studies. We considered the study quality high if the score was 6 points and above out of 9 points [27]. When we scored the adjustment for confounders, age, sex, BMI/weight, smoking status, physical activity, alcohol intake, and total energy intake were considered as primary confounders. Intakes of major foods (e.g., fruits, vegetables, whole grains, red meat) or diet quality (e.g., alternative healthy eating index) were considered as secondary confounders.

We used relative risks (RRs) and 95%CIs to assess risk of mortality across studies, and treated hazard ratios (HRs) and odds ratios (ORs) as equivalent to RRs. We used servings of ASB intake to harmonize exposures among studies and quantify the amount of ASB intake in the meta-analysis. For the four studies that reported ASB consumption in milliliters [12, 13, 16, 21], we converted these measurements to servings of ASB, assuming 1 serving to be 355 milliliters. We determined intake levels using the median or mean of each ASB intake category when available, or the midpoint between the lower and upper boundaries of each category of intake. If the highest category was open-ended, we estimated intake by multiplying the lower boundary of that category by 1.5 [28].

First, we estimated pooed RRs comparing highest with lowest ASB intake using random-effect meta-analysis by combing the risk estimates from the highest category of intake compared with the lowest category reported in each selected study. Second, we conducted a linear dose-response meta-analysis for each one serving increase of ASB intake in relation to mortality. This was done by first calculating study-specific slope lines for the studies that did not reported associations of each one serving of ASB intake, and then which were combined with studies where the slopes were directly reported, to obtain an overall average slope [29]. Third, to test potential non-linearity of the association between ASB intake and mortality, we applied a two-stage random effects dose-response meta-analysis. We modelled ASB intake by using restricted cubic splines with 3 knots at the 10th, 50th, and 90th percentiles of ASB intake. Using the Orsini method [30], the correlations within each set of reported risk estimates were taken into account by a generalized least squares trend estimation method. And then a restricted maximum likelihood method was applied to combine the specific estimates of these studies in a multivariate random effects meta-analysis. We estimated the P value of non-linearity by null hypothesis testing, in which we assumed that coefficient of the second spline was equivalent to zero.

Between-study heterogeneity in the pooled estimates was assessed using the I^2^ statistic (I^2^: 0–40%, not be important heterogeneity; 30–60%, moderate heterogeneity; 50–90%, substantial heterogeneity; 75–100%, considerable heterogeneity) [31].

A secondary meta-analysis was conducted in a subgroup of cohort studies to summarize the associations between substation of SSB with ASB and all-cause and CVD mortality to explore whether ASB were as unhealthy as SSB.

### Sensitivity analyses

To further explore potential sources of heterogeneity among studies and test the robustness of the associations, we conducted several sensitivity analyses. First, we performed predefined subgroup analyses stratified by age, sex, region, follow-up duration, number of participants, number of events, level of ASB intake, adjustment for total energy intake, and dietary assessment method. Second, we conducted Begg’s test and Egger’s test as well as visually inspected the funnel plot to test publication bias. Finally, we examined the influence of individual studies on the overall risk estimate, which was investigated by omitting one study at a time from the meta-analysis and recalculating the RR.

We used Stata version 17.0 (StataCorp, College Station, TX) to perform statistical analyses for the meta-analysis.

### Assessment of the quality of evidence

We used the NutriGrade scoring system for meta-analyses of cohort studies to assess the overall quality of evidence supporting the association of ASB intake and mortality risk [25]. This scoring system includes the following eight items: (1) risk of bias (ROB), study quality, and study limitations (0–2 points); (2) precision (0–1 point); (3) heterogeneity (0–1 point); (4) directness (0–1 point); (5) publication bias (0–1 point); (6) funding bias (0–1 point); (7) effect size (0–2 points); and (8) dose-response (0–1 point) [25]. Per the NutriGrade scoring system, four ranks for quality of meta-analysis are recommended: ≥ 8 points, high quality; 6-7.99 points, moderate quality; 4-5.99 points, low quality; and 0-3.99, very low quality [25]. The assessment was conducted in duplicate independently by two authors (CW and KW), and any disagreements or discordances in the scoring were resolved through discussion.

## Results

### Study selection and characteristics

**Fig. 1:**
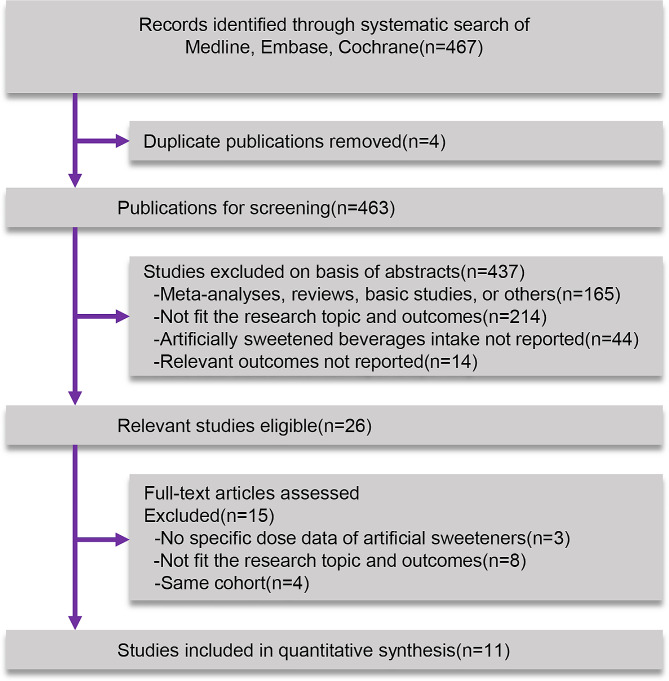
Flow chart of study selection

467 unique records were identified, 441 of which were excluded after reviewing the titles and abstracts, resulting in the identification of 26 publications for full-text review. After full-text screening, 15 articles were excluded. Finally, 11 articles were included in the systematic review and meta-analysis. These studies encompassed a total of 2,196,503 participants and 235,609 recorded deaths, of which 17,263 from CVD and 161,151 from cancer (Fig. 1).

Table 1 and Supplementary Table 2 list specific characteristics of the included prospective studies. These studies ranged in sample size from 13,624 to 934,777 participants, with a mean age range from 46.7 to 73.8 years, and a median/mean follow-up period from 7.0 to 28.9 years. Of the 11 studies, seven were conducted in the US [11, 13–15, 17, 22, 23] and four in Europe [12, 16, 21, 24]. Two studies focused exclusively on women [14, 15], and nine on men and women together [11–13, 16, 17, 21–24]. Eight studies measured dietary data using food questionnaires [11–15, 17, 23, 24], while the other three applied 24-h dietary recalls [16, 21, 22].

**Table 1:** Characteristics of the studies included in the meta-analysis

Author, year	Study population	Country	Participants, *n*	Follow-up (years)	Number of death cases, *n*
Naomi et al., 2023	Lifelines Cohort Study	Netherlands	118,707	Median: 9.8	All-cause mortality: 2,852
McCullough et al., 2022	Cancer Prevention Study-II (CPS-II) prospective cohort	United States	934,777	Median: 27.7	Cancer mortality: 135,093
Liu et al., 2022^a^	UK Biobank	United Kingdom	171,616	Median: 7.0	All-cause mortality: 1,087 CVD mortality: 252 Cancer mortality: 564
Zhang et al., 2021	National Health and Nutrition Examination Survey	United States	31,402	Mean: 7.9	All-cause mortality: 3,878 CVD mortality: 676 Cancer mortality: 883
Anderson et al., 2020^a^	UK Biobank	United Kingdom	161,415	Mean: 7.0	All-cause mortality: 2,311
Keller et al., 2020	The Harvard Pooling Project (HPP) of Diet and Coronary Disease	United States	284,345	Median: 8.2	CHD mortality:1,630
Mullee et al., 2019	The European Prospective Investigation into Cancer and Nutrition (EPIC)	10 European Countries	451,743	Mean: 16.4	All-cause mortality: 29,045 CVD mortality: 5,867 Cancer mortality: 12,231
Malik et al., 2019	The Health Professional’s Follow-up study (HPFS, from 1986 to 2014) and the Nurses’ Health study (NHS, from 1980 to 2014)	United States	118,363	Mean: 28.9	All-cause mortality: 36,436 CVD mortality: 7,896 Cancer mortality: 12,380
Mossavar-Rahmani et al., 2019^b^	The Women’s Health Initiative Observational Study	United States	71,926	Mean: 11.9	All-cause mortality: 12,978
Vyas et al., 2015^b^	The Women’s Health Initiative Observational Study	United States	59,614	Mean: 8.7	CVD mortality: 942
Paganini-Hill et al., 2007	The Leisure World Cohort Study	United States	13,624	Mean: 13.2	All-cause mortality: 11,386

^a^The study by Liu et al. in the UK Biobank was included in the meta-analysis for CVD and cancer mortality but not all-cause mortality, while the study by Anderson et al., also from the UK Biobank, including more participants and examining general artificial sweetened beverages and all-cause mortality but not cause-specific mortality was included in the meta-analysis for all-cause mortality. ^b^The study by Vyas et al. in The Women’s Health Initiative Observational Study was included in the meta-analysis for CVD mortality, while the Study by Mossavar-Rahmani et al. also from The Women’s Health Initiative Observational Study including more participants but only examining the associations with all-cause mortality, was included in the meta-analysis for all-cause mortality

Seven studies adjusted for all primary and secondary confounders [11, 12, 16, 21–24] (Supplementary Table 3). Supplementary Table 4 presents the assessment of ROB of included studies, as assessed using the Newcastle-Ottawa Scale. All the 11 studies scored at least 6 points, suggesting a low ROB and high study quality.

For the current study, ten studies were included in the highest vs. lowest ASB intake meta-analysis [11–16, 21–24], ten in per-serving/day of ASB meta-analysis [11, 12, 15–18, 21–24], and nine in the dose-response meta-analysis [11–13, 15, 16, 21, 22, 24].

### Associations of ASB consumption with mortality

**Fig. 2:**
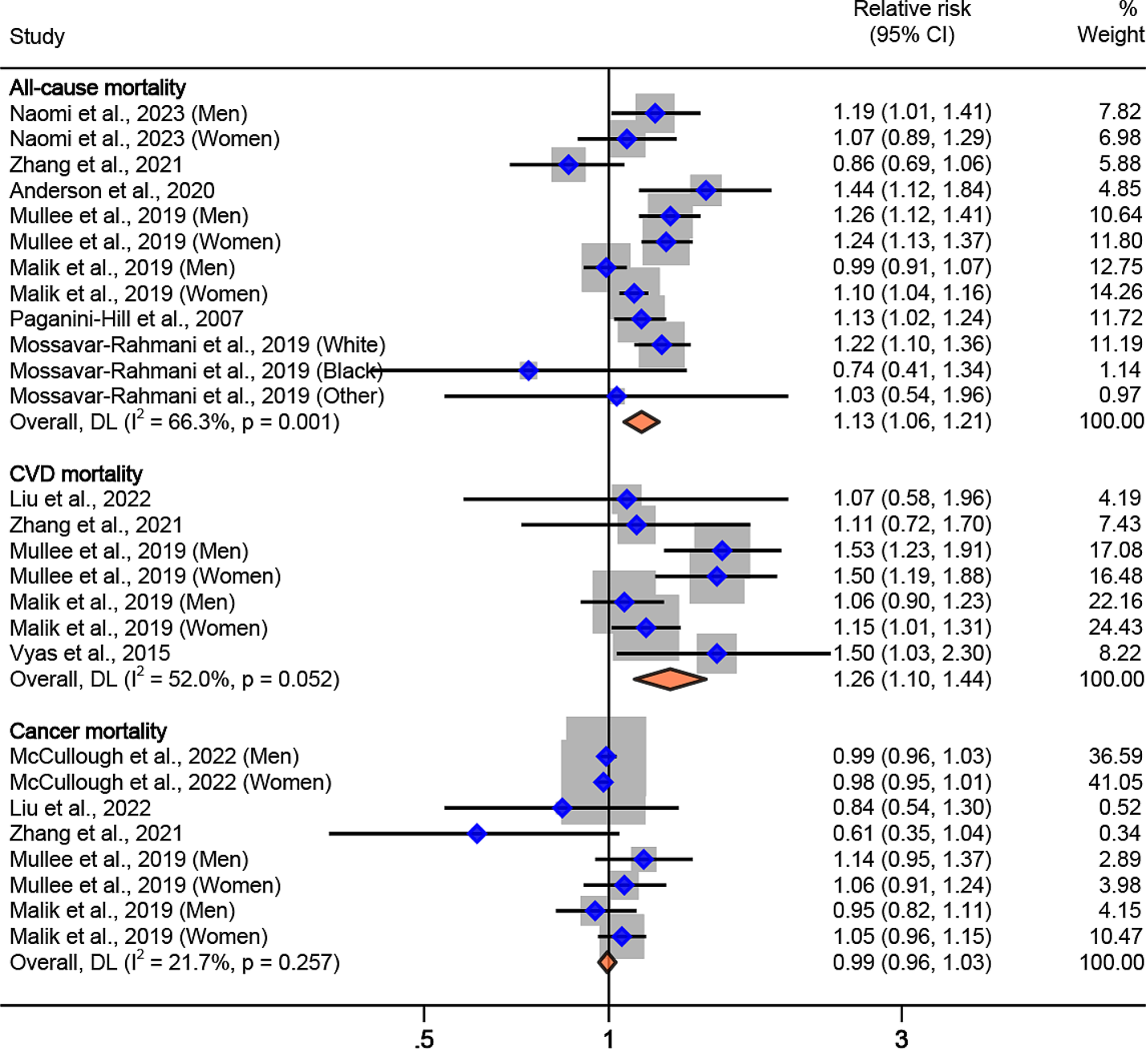
Association of artificially sweetened beverage consumption with all-cause, CVD, and cancer mortality for highest vs. lowest intake, using random-effects meta-analysis. Weights of each of the estimates are represented by the size of the square. Blue diamond represents the individual estimate effects and black lines represent the 95% confidence interval. The x axis is the relative risk. The pooled effect estimates, and 95% confidence intervals are represented by the diamond. I^2^ refers to the proportion of heterogeneity between studies

**Fig. 3:**
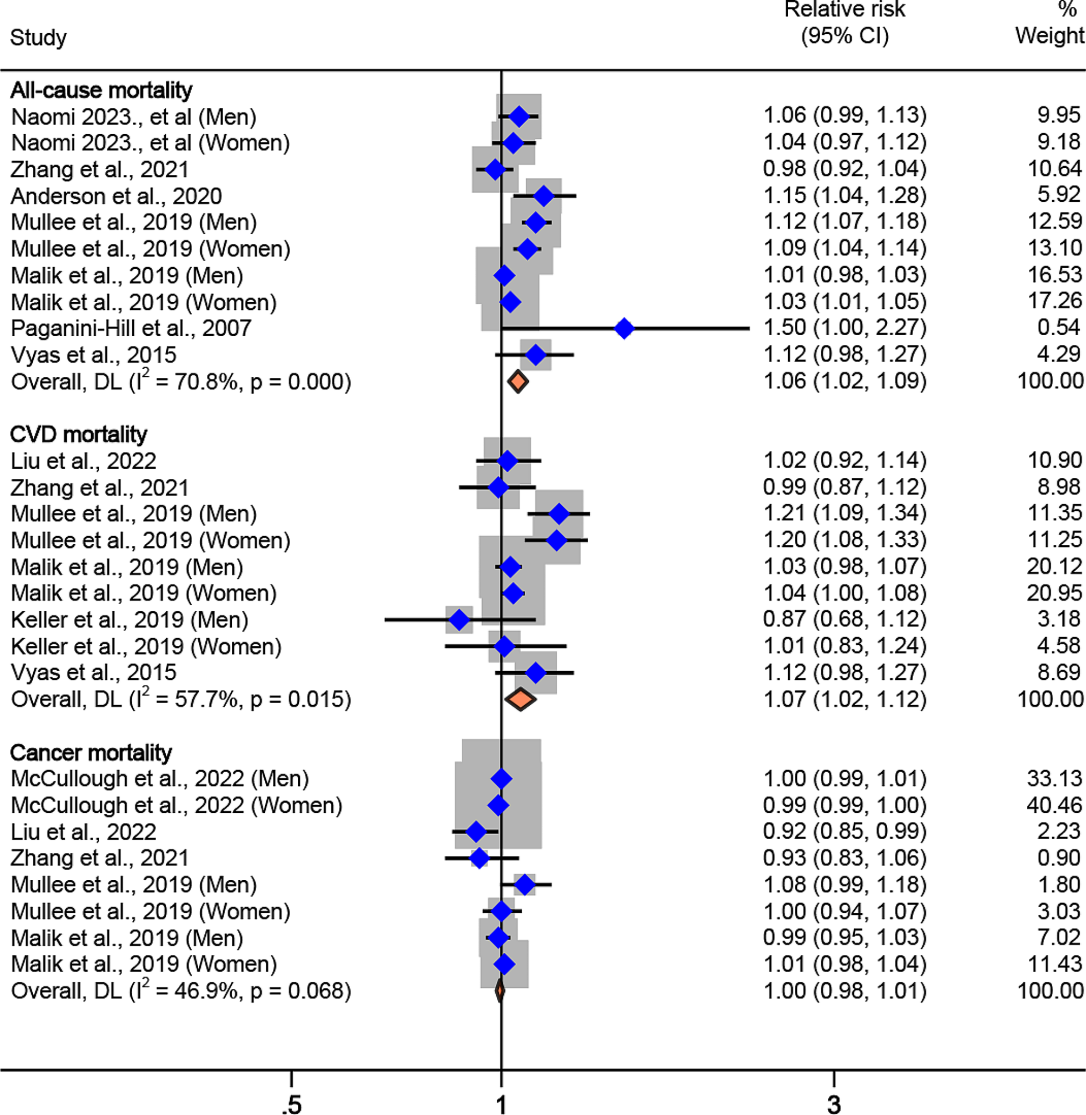
Association of artificially sweetened beverage consumption with all-cause, CVD, and cancer mortality, for 1 serving per day, using random-effects meta-analysis. Weights of each of the estimates are represented by the size of the square. Blue diamond represents the individual estimate effects and black lines represent the 95% confidence interval. The x axis is the relative risk. The pooled effect estimates, and 95% confidence intervals are represented by the diamond. I^2^ refers to the proportion of heterogeneity between studies

Our analysis showed that the pooled RR for mortality for the highest vs. the lowest level of ASB consumption was 1.13 (95%CI: 1.06, 1.21; I^2^ = 66.3%, P_*heterogeneity*_=0.001) for all-cause mortality, 1.26 (95%CI: 1.10, 1.44; I^2^ = 52.0%, P_*heterogeneity*_=0.05) for CVD mortality, and 0.99 (95%CI: 0.96, 1.03; I^2^ = 21.7%, P_*heterogeneity*_=0.26) for cancer mortality (Table 2; Fig. 2). For every increase of one serving per day in ASB intake, the pooled RR was 1.06 (95%CI: 1.02, 1.09; I^2^ = 70.8%, P_*heterogeneity*_ <0.001) for all-cause mortality, 1.07 (95%CI: 1.02, 1.12; I^2^ = 57.7%, P_*heterogeneity*_=0.02) for CVD mortality, and 1.00 (95%CI: 0.98, 1.01; I^2^ = 46.9%, P_*heterogeneity*_=0.07) for cancer mortality (Table 2; Fig. 3).

**Table 2:** Meta-analysis of multivariable relative risks for the associations between artificially sweetened beverage consumption and mortality using random-effects^a^

ASB consumption	Highest vs. lowest intake						Per one serving per day increase					
	Risk estimates (*n*)	Participants (*n*)	Cases (*n*)	Pooled Relative Risk (95% CI)	I^2^ (%)^b^	Risk estimates (*n*)		Participants (*n*)	Cases (*n*)	Pooled Relative Risk (95% CI)	I^2^ (%)^b^	
All-cause mortality	12	967,180	98,886	1.13 (1.06, 1.21)	66.3	10		954,868	90,345	1.06 (1.02, 1.09)	70.8	
CVD mortality	7	832,738	15,633	1.26 (1.10, 1.44)	52.0	9		1,117,083	17,263	1.07 (1.02, 1.12)	57.7	
Cancer mortality	8	1,707,901	161,151	0.99 (0.96, 1.03)	21.7	8		1,707,901	161,151	1.00 (0.98, 1.01)	46.9	

^a^ Pooled relative risks are from random-effects meta-analyses

^b^ Refers to the proportion of heterogeneity between studies

**Fig. 4:**
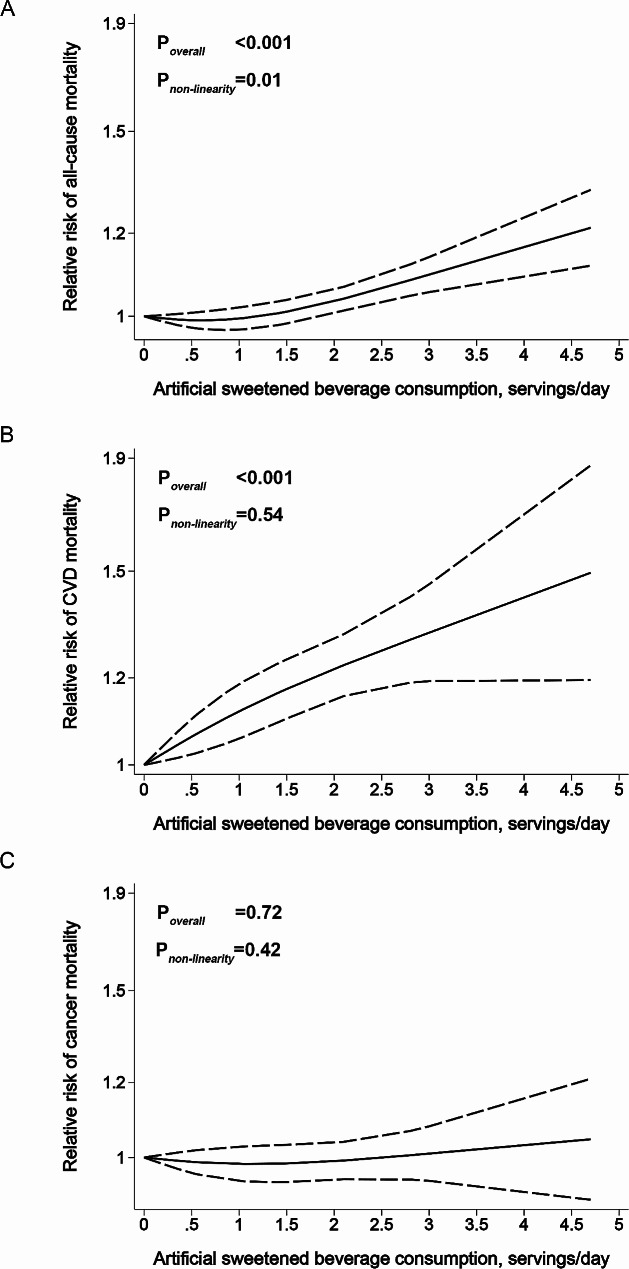
Dose-response association of artificially sweetened beverage consumption with all-cause, CVD, and cancer mortality, using restricted cubic spline

No significant non-linear association for ASB intake and CVD mortality was seen (P_*non−linearity*_=0.54, P_*overall*_<0.001), meaning that an unfavorable linear dose-response association was seen for CVD mortality, irrespective of the specific dose level. However, a nonlinear association was observed for all-cause mortality (P_*non−linearity*_=0.01, P_*overall*_<0.001) (Fig. 4). Specifically, consuming above one serving per day showed an unfavorable dose-response relationship with all-cause mortality. There was no dose-response relationship for cancer mortality.

**Fig. 5:**
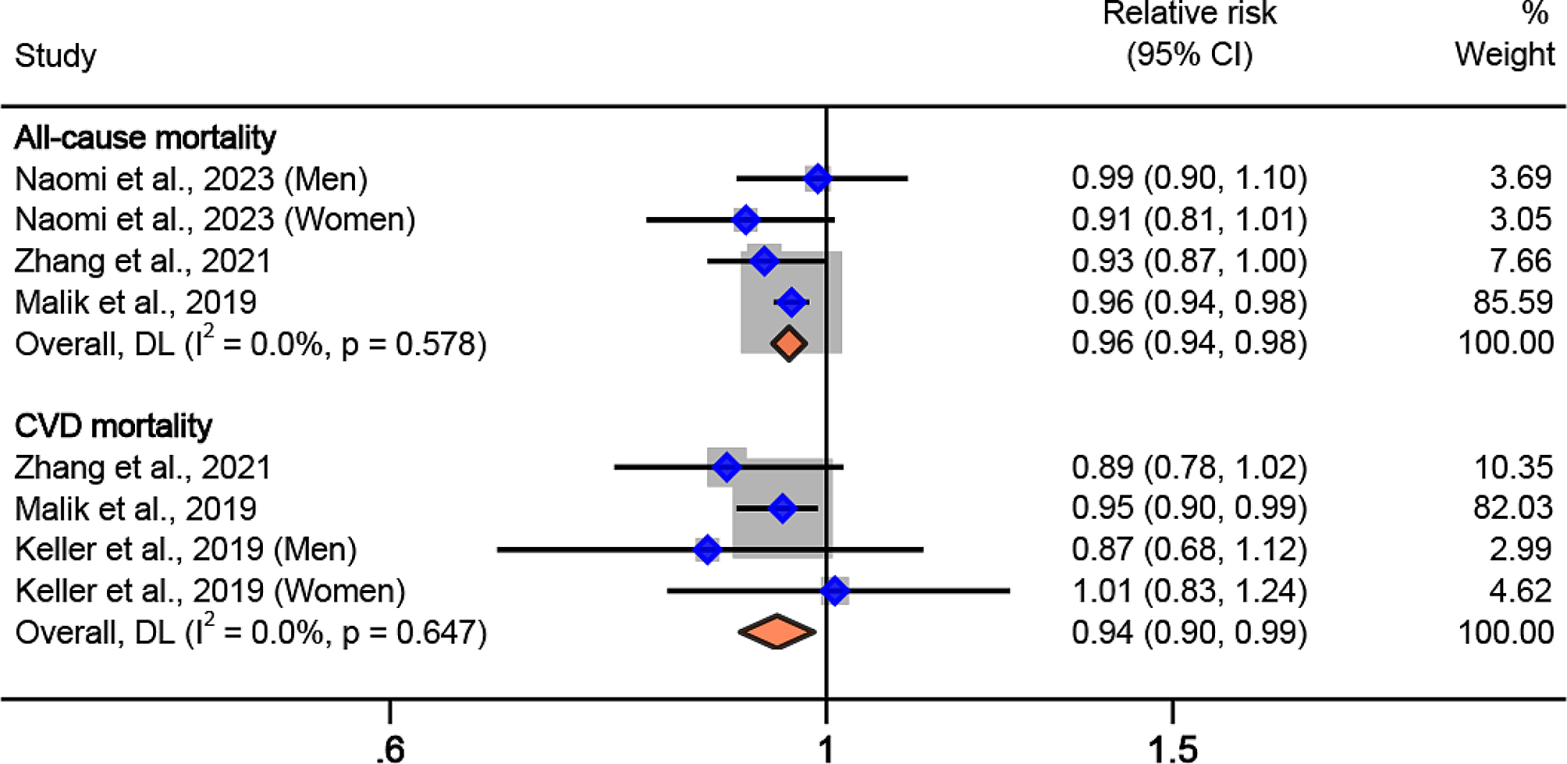
Hazard ratio of all-cause, CVD, and cancer mortality when substituting 1 serving/d of sugar sweetened beverages with equivalent amounts of artificially sweetened beverages, using random-effects meta-analysis. Weights of each of the estimates are represented by the size of the square. Blue diamond represents the individual estimate effects and black lines represent the 95% confidence interval. The x axis is the relative risk. The pooled effect estimates, and 95% confidence intervals are represented by the diamond. I^2^ refers to the proportion of heterogeneity between studies

The secondary meta-analysis indicated substitution of SSB with ASB in relation to a lower risk of all-cause and CVD mortality. The pooled RRs and 95%CIs for substituting 1 serving/d of SSB with equivalent amounts of ASB were 0.96 (0.94, 0.98) (I^2^ = 0.0%, P_*heterogeneity*_=0.58) for all-cause mortality and 0.94 (0.90, 0.99) (I^2^ = 0.0%, P_*heterogeneity*_=0.65) for CVD mortality (Fig. 5).

The associations between ASB consumption and risk of all-cause, and cancer mortality did not differ by age, region, sex, number of cases, number of participants, duration of follow-up, dietary assessment methods, level of ASB intake, and adjustment for total energy (All P_*interaction*_ values ≥ 0.05) (Supplementary Table 5). Yet, a stronger association of ASB consumption with CVD mortality was observed in Europe than in USA (P_*interaction*_ =0.01), and for the populations with a lower level of ASB intake than those with a higher level of ASB intake (P_*interaction*_ =0.03), while the associations of ASB with CVD mortality were consistent across these above-mentioned characteristics except for region and level of ASB intake (Supplementary Table 5).

The Begg’s and Egger’s tests and the visual examination of the funnel plot (Supplementary Fig. 1) provided no evidence of publication bias. In addition, no single study significantly caused heterogeneity (Supplementary Fig. 2).

Finally, according to the NutriGrade scoring system, the quality of meta-evidence was moderate for ASB consumption in relation to all-cause and CVD mortality, while the quality was low for ASB consumption with cancer mortality (Supplementary Table 6).

## Discussion

### Main findings

Our systematic review and meta-analysis demonstrated a higher consumption of ASB in relation to higher risks of all-cause and CVD mortality, whereas no relationship of ASB with cancer mortality was observed. Compared with the participants in the lowest category of ASB intakes, those in the highest category had a 13% higher risk of premature death from any cause, and a 26% higher risk of CVD mortality. Each one additional serving increase in ASB consumption was associated with 6% and 7% higher risk for all-cause and CVD mortality, respectively. In a dose-response meta-analysis, we also observed a linear association of ASB consumption with CVD mortality, with a non-linear positive association of ASB with all-cause mortality. Despite this, substitution of SSB with ASB was associated with a lower risk of all-cause and CVD mortality. Various sensitivity analyses and subgroups analyses demonstrated the robustness of the pooled associations. Per NutriGrade, quality of the overall evidence was scored moderate for CVD mortality and all-cause mortality.

### Compared with previous studies

The potential health effects of ASB are a topic of extensive discussion. Previous meta-analyses of short-term RCTs demonstrated that low/no-calorie sweeteners may have modest benefits on measures of obesity (e.g., body weight, BMI, fat mass, and waist circumference), blood glucose, and blood pressure [7, 32]. Yet, long-term effects of artificial sweeteners or their beverages on cardiometabolic diseases have not been explored in clinical trials and may not be feasible. However, meta-analyses of observational studies, similar to ours, have observed harmful relationships of ASB consumption in relation to all-cause and CVD mortality [18–20].

By incorporating recently published large cohort studies into our current meta-analysis, we reaffirmed the harmful associations of ASB intake with all-cause and CVD mortality in observational studies. Additionally, in contrast with a previous dose-response meta-analysis that suggested J-shape relationships, we detected a linear dose-response relationship with CVD mortality. We did observe a non-linear dose-response relationship with all-cause mortality with an increased risk only at ASB intake levels above 1 serving of ASB per day. It is important to note that the differences in the observed dose-response relationships may be attributable to differences in the included studies and their population characteristics, although we do not see any obvious characteristics that can explain this. More importantly, the previous meta-analysis had a low overall quality of meta-evidence, whereas our meta-analysis, with more studies included, was rated as moderate as per the NutriGrade scoring system. Furthermore, the previous meta-analyses were also limited by lack of specific evidence regarding the associations of the comparisons of ASB and other drinks (e.g., SSB) with mortality. To address the limitation, we pooled the associations between substitution of SSB with ASB and mortality in a secondary meta-analysis and observed the inverse associations, suggesting that ASB could be a suitable replacement for SSB among habitual high SSB consumers. However, we could not exclude the possibility that the weak inverse associations of substitution of SSB with ASB are likely due to residual confounding, as individuals at high risks (e.g., overweight persons) may be more likely to choose ASB in replacement of SSB to improve cardiometabolic health. In addition, as very few cohorts, to date, have examined the associations of the comparisons of ASB with a few healthy drinks (e.g., water, tea) with mortality, we could not further summarize the associations based on the comparisons of ASB with these healthy drinks. Yet, it is of critical importance to explore the associations of substitutions between ASB and healthy drinks to better understand the potential impact of ASB on health. Thus, more cohort studies are needed to explore these associations.

### Potential mechanisms

The pathways behind the harmful associations of ASB intakes with mortality remain unclear, but evidence from human studies has indicated higher intake of artificial sweeteners in relation to higher level of cardiometabolic risk factors, such as obesity, glucose and hypertriglyceridemia, and higher risk of CVD [33–36]. For example, Suez et al. observed that artificial sweeteners impair glycemic responses through altering gut microbiota in a short time RCT [36]. Moreover, animal experiments have shown that artificial sweeteners may impair secretion of insulin by lowing release of glucagon-like peptide-1, resulting in hyperglycemia [37]. Also, artificial sweeteners may influence insulin secretion and glucose metabolism via involvement of intestinal sweet taste receptors [38]. In addition, Basson and colleagues have indicated artificial sweetener consumption in relation to higher level of increased inflammation [39], a risk factor for CVD. Further studies are needed to explore the mechanisms underlying associations of ASB or artificial sweeteners with CVD risk.

### Strengths and limitations

This is the largest and most comprehensive meta-analysis of prospective cohorts examining the associations between ASB intake and mortality up to date, with 2,196,503 participants, which was more than twice that of the previous meta-analyses. Further, this is also the first to pool the associations of substitution of SSB with ASB with mortality, and assess the quality of meta-analysis results using the NutriGrade scoring system. These results of our meta-analyses could provide valuable insights into formulating dietary guidelines.

However, several limitations should be considered. Firstly, dietary intake, including ASB intake was assessed using self-reported food frequency questionnaires, and 24-hour diet recalls, and thus measurement errors and misclassifications were inevitable. Furthermore, we could not distinguish specific types of artificial sweeteners contained in the ASB, while health effects of artificial sweeteners may differ per types of sweetener [33]. However, misclassifications from prospective cohorts tend to be nondifferential, which would be likely to dilute true associations and potentially lead to an underestimation of the true effect size. Secondly, due to limited studies for ASB consumption and other non-CVD and non-cancer mortality, we could not further summarize the associations with other non-CVD and non-cancer mortality. Thirdly, these results should be interpreted cautiously because of moderate quality of meta-evidence for ASB with all-cause and CVD mortality, and low quality for cancer mortality, which were assessed using NutriGrade Scoring system. Fourth, several of the meta-analysis presented significant heterogeneity, which could be due to the varying populations, the different levels of ASB intake, and definitions of ASB across the studies. Indeed, we observed a stronger association of ASB consumption with CVD mortality among European populations rather than US populations, and the average levels of ASB intake in Europe were also lower than those in the US. Further, a few studies only reported the subgroup-specific associations by sex or race and these subgroup-specific associations were directly included in the overall meta-analysis [11, 12, 14, 23, 24], which might lead to the overestimate of the overall heterogeneity due to the heterogeneity between the subgroup-specific associations from the same study. Yet, most of I^2^ values between the subgroups by sex or race from the same study for ASB intake and all-cause and cause-specific mortality were minor (≤ 30%, not important heterogeneity) [11, 12, 14, 23, 24], only I^2^ values between the subgroups by sex from the study by Malik et al. [11] for all-cause mortality and from the study by McCullough et al. [23] for cancer mortality were 77.6% and 67.7%, respectively, which may not significantly influence the overall heterogeneity. Finally, we could not establish causality due to the observational nature of the studies.

## Conclusions

Our study suggests that higher ASB intakes are associated with increased risks of all-cause and CVD mortality, while ASB could be used to replace SSB among habitual SSB consumers. These findings should be interpreted cautiously due to the limitations and moderate quality of the meta-analysis. It is imperative that further high-quality research is conducted to confirm our findings and to explore the long-term impact of ASB intake on mortality. Despite these limitations, our study adds to the growing body of evidence on the potential health risks associated with ASB consumption and could inform future dietary guidelines and public health interventions.

### Electronic supplementary material

Below is the link to the electronic supplementary material.

Supplementary Material 1

## Data Availability

No datasets were generated or analysed during the current study.

## Abbreviations

ASB: Artificially sweetened beverages
RRs: Risk ratios
95%CIs: 95% confidence intervals
WHO: World Health Organization
SSB: Sugary sweetened beverages
RCTs: Randomized controlled trials
ROB: Risk of bias
CVD: Cardiovascular disease
HRs: Hazard ratios
ORs: Odds ratios
