# Supplementary material for: Artificially sweetened beverage consumption and all-cause and cause-specific mortality: an updated systematic review and dose-response meta-analysis of prospective cohort studies
Source: Nutr J. 2024 Jul 31;23:86. doi: 10.1186/s12937-024-00985-7 (PMC11290234; doi:10.1186/s12937-024-00985-7)

**Online Supplementary Tables and Figures**

**Artificially sweetened beverage consumption and all-cause and cause-specific mortality: an updated systematic review and dose-response meta-analysis of prospective cohort studies**

Zhangling Chen, Cheng Wei, Sander Lamballais, Kang Wang, Yuchan Mou, Yichao Xiao, Fei Luo, Wichor Bramer, Trudy Voortman, Shenghua Zhou

Table of content

[Supplementary Table 1: Search strategy 2](#_Toc170143330)

[Supplementary Table 2: Characteristics of the included studies on the associations of intake of artificially sweetened beverages and risk of all-cause and cause-specific mortality. 3](#_Toc170143331)

[Supplementary Table 3: Inclusion of primary and secondary confounders in adjusted models of included studies 8](#_Toc170143332)

[Supplementary Table 4: Newcastle-Ottawa scale score for each included study 9](#_Toc170143333)

[Supplementary Table 5: Pre-specified subgroup meta-analyses of the association between artificially sweetened beverage consumption and risk of all-cause and cause-specific mortality, using random-effects models. 10](#_Toc170143334)

[Supplementary Table 6: Assessment of the quality of evidence on the relationship between artificially sweetened beverage consumption and all-cause and cause-specific mortality using the NutriGrade scoring system. 13](#_Toc170143335)

[Supplementary Figure 1: Funnel plot for assessment of publication bias for association between artificially sweetened beverage consumption and risk of all-cause and cause-specific mortality. 14](#_Toc170143336)

[Supplementary Figure 2: Forest plot of influence analysis for the association between artificially sweetened beverage consumption and risk of all-cause and cause-specific mortality. 14](#_Toc170143337)

# Supplementary Table 1: Search strategy

| **Database** | **Search term** |
| --- | --- |
| **Medline All** | (Artificially Sweetened Beverages/ OR ((Sweetening Agents/ OR Aspartame/ OR Saccharin/) AND (Beverages/ OR Drinking Behavior/)) OR (((artificial* OR low-calor* OR no-calor*) ADJ3 sweeten* AND (coke OR soda OR drink OR drinks OR beverage*)) OR ((diet OR low-calor* OR light OR acesulfam* OR aspartam* OR saccharin* OR steviosid* OR nonnutritive-sweet* OR non-nutritive-sweet*) ADJ3 (soft-drink* OR coke OR soda OR drink OR drinks OR beverage*))).ab,ti.) AND (Mortality/ OR Cause of Death/ OR (mortalit* OR (cause ADJ3 death)).ab,ti.) NOT (exp animals/ NOT humans/) |
| **embase.com** | ('artificially sweetened beverage'/de OR (('sweetening agent'/de OR 'low calorie sweetener'/de OR 'acesulfame'/de OR 'aspartame'/de OR 'aspartame derivative'/de OR 'saccharin'/de OR 'saccharin derivative'/de OR 'saccharin sodium'/de OR 'stevioside'/de OR 'nonnutritive sweetener'/de) AND (beverage/exp OR 'drinking behavior'/de)) OR (((artificial* OR low-calor* OR no-calor*) NEAR/3 sweeten* AND (coke OR soda OR drink OR drinks OR beverage*)) OR ((diet OR low-calor* OR light OR acesulfam* OR aspartam* OR saccharin* OR steviosid* OR nonnutritive-sweet* OR non-nutritive-sweet*) NEAR/3 (soft-drink* OR coke OR soda OR drink OR drinks OR beverage*))):ab,ti) AND (mortality/exp OR 'mortality risk'/de OR 'cause of death'/de OR (mortalit* OR (cause NEAR/3 death)):ab,ti) NOT ([animals]/lim NOT [humans]/lim) |
| **Web of Science** | TS=(((((artificial* OR low-calor* OR no-calor*) NEAR/2 sweeten* AND (coke OR soda OR drink OR drinks OR beverage*)) OR ((diet OR low-calor* OR light OR acesulfam* OR aspartam* OR saccharin* OR steviosid* OR nonnutritive-sweet* OR non-nutritive-sweet*) NEAR/2 (soft-drink* OR coke OR soda OR drink OR drinks OR beverage*)))) AND ((mortalit* OR (cause NEAR/2 death)))) NOT DT=(Meeting Abstract OR Meeting Summary) AND LA=(english) |
| **Cochrane CENTRAL** | ((((artificial* OR low-calor* OR no-calor*) NEAR/3 sweeten* AND (coke OR soda OR drink OR drinks OR beverage*)) OR ((diet OR low-calor* OR light OR acesulfam* OR aspartam* OR saccharin* OR steviosid* OR nonnutritive-sweet* OR non-nutritive-sweet*) NEAR/3 (soft-drink* OR coke OR soda OR drink OR drinks OR beverage*))):ab,ti) AND ((mortalit* OR (cause NEAR/3 death)):ab,ti) |

# Supplementary Table 2: Characteristics of the included studies on the associations of intake of artificially sweetened beverages and risk of all-cause and cause-specific mortality.

| **Author, year** | **Study population** | **Country** | **Participants, n** | **Age (years)** | **Follow-up (years)** | **Assessment of diet** | **Number of death cases** | **Ascertainment of cases** | **Statistical model** | **Consumption**  **servings/day** | **Categories of exposure** | **Relative risks (95% CI)** | **Covariates in multivariable model** |
| --- | --- | --- | --- | --- | --- | --- | --- | --- | --- | --- | --- | --- | --- |
| Naomi et al., 2023 | Lifelines Cohort Study | Netherlands | 118,707  Men:47,943  Women:70,764 | 45 ± 13 | Median:  9.8 | FFQ | All-cause mortality  2,852 | National Personal Records Database | Cox proportional hazards regression model | Median: 0.10 | **Men**  Consumption  0 serving/d  >2 servings/wk  >2 to <7 servings/wk  >=1 servings/d  **Women**  Consumption  0 serving/d  >2 servings/wk  >2 to <7 servings/wk  >=1 servings/d | **All-cause mortality**  1.00 (reference)  0.93 (0.81-1.06)  0.99 (0.86-1.14)  1.19 (1.01-1.41)  1.00 (reference)  0.93 (0.81-1.07)  0.91 (0.78-1.07)  1.07 (0.89-1.29) | Age, sex, education level, alcohol consumption, smoking status, moderate physical activity, sedentary behavior, baseline BMI, consumptions of grain, potatoes, vegetables, fruit, meat and processed meat, coffee, tea, legumes, nuts, fats and oils, sugary foods, mutual adjustment for other beverages (SSB, LNCB, and fruit juice), and energy intake |
| McCullough et al., 2022 | Cancer Prevention Study-II (CPS-II) prospective cohort | United States | 934,777  Men:416,313  Women:518,464 | ≥28 | Median:  27.7 | FFQ | Cancer mortality  135,093 | Death certificates | Cox proportional hazards regression model | Mean: 0.25 | **Men**  Consumption  0 drinks/d  <1 drinks/d  1 drink/d  2+ drinks/d  **Women**  Consumption  0 drinks/d  <1 drinks/d  1 drink/d  2+ drinks/d | **Cancer mortality**  1.00 (reference)  0.98 (0.95-1.00)  0.99 (0.95-1.02)  0.99 (0.96-1.03)  1.00 (reference)  0.97 (0.95-0.99)  0.98 (0.96-1.01)  0.98 (0.95-1.01) | Age, sex, race/ethnicity, smoking at 1982 baseline, marital status, education, red and processed meat consumption, fruit and vegetable consumption, alcohol consumption, and sugar-sweetened beverage consumption. |
| Liu et al., 2022^a^ | UK Biobank | United Kingdom | 171,616  Men:76,527  Women:95,089 | 37-73 | Median:7 | Oxford WebQ | All-cause mortality  1,087  CVD mortality  252  Cancer mortality  564 | Death certificates | Cox proportional hazards regression model | Mean: 0.21 | Consumption  0 drinks/d  > 0-1.5 drinks/d  > 1.5-2.5 drinks/d  > 2.5-3.5 drinks/d  > 3.5-4.5 drinks/d  > 4.5 drinks/d  Consumption  0 drinks/d  > 0-1.5 drinks/d  > 1.5-2.5 drinks/d  > 2.5-3.5 drinks/d  > 3.5-4.5 drinks/d  > 4.5 drinks/d  Consumption  0 drinks/d  > 0-1.5 drinks/d  > 1.5-2.5 drinks/d  > 2.5-3.5 drinks/d  > 3.5-4.5 drinks/d  > 4.5 drinks/d | **All-cause mortality**  1.00 (reference)  0.95 (0.75-1.21)  0.94 (0.73-1.19)  0.88 (0.67-1.16)  0.65 (0.45-0.92)  0.89 (0.65-1.2)  **CVD mortality**  1.00 (reference)  1.32 (0.84-2.07)  0.88 (0.51-1.52)  1.24 (0.73-2.1)  1.06 (0.57-1.98)  1.07 (0.58-1.96)  **Cancer mortality**  1.00 (reference)  0.97 (0.7-1.35)  0.87 (0.61-1.23)  0.76 (0.5-1.15)  0.51 (0.3-0.89)  0.84 (0.54-1.3) | Age, sex, TDI, education level, ethnicity, smoking status, pack-years of smoking, physical activity level, body mass index, waist circumference, hypertension, diabetes, depression, family history of CVD, family history of cancer, long-standing illness, cholesterol-lowering drug use, blood pressure drug use, vitamin and mineral supplement use, and intake of total energy, total sugar, fresh fruit, vegetables, red meat, processed meat, alcohol, tea, milk, SSBs, and ASBs. |
| Zhang et al., 2021 | National Health and Nutrition Examination Survey | United States | 31,402  Men:15,842  Women:15,560 | ≥20 | Mean:7.9 | 24-h dietary recalls | All-cause mortality  3,878  CVD mortality  676  Cancer mortality  883 | National Death Index | Cox proportional hazards regression model | Mean: 0.37 | Consumption  0 servings/d  > 0 to <1 servings/d  1 to <2 servings/d  >=2 servings/d  Consumption  0 servings/d  > 0 to <1 servings/d  1 to <2 servings/d  >=2 servings/d  Consumption  0 servings/d  > 0 to <1 servings/d  1 to <2 servings/d  >=2 servings/d | **All-cause mortality**  1.00 (reference)  0.84 (0.72-0.97)  0.89 (0.77-1.04)  0.86 (0.69-1.06)  **CVD mortality**  1.00 (reference)  0.64 (0.45-0.91)  0.66 (0.45-0.98)  1.11 (0.72-1.70)  **Cancer mortality**  1.00 (reference)  0.91 (0.64-1.29)  1.18 (0.84-1.66)  0.61 (0.35-1.04) | Age, sex, family income-poverty ratio, self-reported race, education level, marital status, alcohol consumption, cigarette smoking, and leisure-time physical activity; BMI, prevalent high cholesterol level, hypertension, and diabetes, and history of CVD and cancer; HEI-2015, total daily energy intakes, and intakes of SSBs and ASBs. |
| Anderson et al., 2020^a^ | UK Biobank | United Kingdom | 161,415 | 40-69 | Mean:7 | Oxford WebQ | All-cause mortality  2,311 | Death certificates | Cox proportional hazards regression model | Mean: 0.18 | Consumption  0 glasses/d  1 glasses/d  > 1-2 glasses/d  > 2 glasses/d | **All-cause mortality**  1.00 (reference)  0.92 (0.81-1.05)  1.13 (0.91-1.39)  1.44 (1.12-1.84) | Sex, age, ethnicity, income, highest qualification, physical activity, sedentary behaviour, total energy intake, body mass index, smoking status, alcohol intake, total sugar intake, total fat intake, fresh fruit intake, vegetable intake, total fibre intake, red meat intake and processed meat intake. |
| Keller et al., 2020^b^ | The Harvard Pooling Project (HPP) of Diet and Coronary Disease | United States | 284,345  Men:68,063  Women:216,282 | ≥35 | Median:8.2 | FFQ | CHD mortality  1,630 | Medical records and death certificates | Cox proportional hazards regression model | None | Consumption  1 serving ASB for SSB  **Men**  **Women** | **CVD mortality**  0.87 (0.68-1.12)  1.01 (0.83-1.24) | Age, smoking, physical activity, education and alcohol, quintiles of cereal fibers; quintiles of trans-fat; quintiles of poly-unsaturated fat/saturated fat ratio, total energy, BMI, baseline hypertension and high cholesterol. |
| Mullee et al., 2019 | The European Prospective Investigation into Cancer and Nutrition (EPIC) | 10 European Countries | 451,743  Men:130,662 Women:321,081 | 50.8±9.8 | Mean:16.4 | Self-administered questionnaires or personal interviews | All-cause mortality  29,045  CVD mortality  5,867  Cancer mortality  12,231 | Record linkages with cancer registries, boards of health, and death indices or through active follow-up (inquiries by mail or telephone to municipal registries or regional health departments or to physicians or hospitals) | Cox proportional hazards regression model | Mean: 0.23 | **Men**  Consumption  0< 1 glass/mo  1 to 4 glasses/mo  > 1 to 6 glasses/wk  1 to <2 glasses/d  >=2 glasses/d  Consumption  0< 1 glass/mo  1 to 4 glasses/mo  > 1 to 6 glasses/wk  1 to <2 glasses/d  >=2 glasses/d  Consumption  0< 1 glass/mo  1 to 4 glasses/mo  > 1 to 6 glasses/wk  1 to <2 glasses/d  >=2 glasses/d  **Women**  Consumption  0< 1 glass/mo  1 to 4 glasses/mo  > 1 to 6 glasses/wk  1 to <2 glasses/d  >=2 glasses/d  Consumption  0< 1 glass/mo  1 to 4 glasses/mo  > 1 to 6 glasses/wk  1 to <2 glasses/d  >=2 glasses/d  Consumption  0< 1 glass/mo  1 to 4 glasses/mo  > 1 to 6 glasses/wk  1 to <2 glasses per/d  >=2 glasses/d | **All-cause mortality**  1.00 (reference)  0.94 (0.88-1.00)  1.06 (0.99-1.13)  1.12 (0.86-1.44)  1.26 (1.12-1.41)  **CVD mortality**  1.00 (reference)  0.81 (0.71-0.94)  1.00 (0.87-1.15)  0.91 (0.51-1.61)  1.53 (1.23-1.91)  **Cancer mortality**  1.00 (reference)  0.99 (0.89-1.09)  1.10 (0.99-1.22)  1.13 (0.76-1.66)  1.14 (0.95-1.37)  **All-cause mortality**  1.00 (Reference)  0.93 (0.88-0.99)  0.97 (0.92-1.03)  0.92 (0.75-1.13)  1.24 (1.13-1.37)  **CVD mortality**  1.00 (Reference)  1.00 (0.88-1.13)  1.01 (0.89-1.15)  1.13 (0.67-1.88)  1.50 (1.19-1.88)  **Cancer mortality**  1.00 (Reference)  0.95 (0.87-1.02)  0.95 (0.88-1.02)  0.83 (0.62-1.11)  1.06 (0.91-1.24) | BMI, physical activity index, educational status, alcohol consumption, smoking status and intensity, ever use of contraceptive pill, menopausal status, ever use of menopausal hormone therapy, intakes of total energy, red and processed meat, fruits and vegetables, coffee, and fruit and vegetable juice, stratified by age (1-year categories), EPIC (European Prospective Investigation into Cancer and Nutrition) center, and sex. |
| Malik et al., 2019 | The Health Professional’s Follow-up study (HPFS, from 1986 to 2014) and the Nurses’ Health study (NHS, from 1980 to 2014) | United States | 118,363  Men:37,716  Women:80,647 | NHS  30 -55  HPFS  40 -75 | Mean: 28.9 | FFQ | **Men**  All-cause mortality  13,004  CVD mortality  3,757  Cancer mortality  4,062  **Women**  All-cause mortality  23,432  CVD mortality  4,139  Cancer mortality  8,318 | Physician review of medical records, autopsy reports, or death certificates | Cox proportional hazards regression model | Mean:  HPFS: 0.38  NHS: 0.32 | **Men**  Consumption  0 < 1 serving/mo  1 to 4 servings/mo  2 to 6 servings/wk  1 to 2 servings/d  >=2 servings/d  Consumption  0 < 1 serving/mo  01 to 4 servings/mo  02 to 6 servings/wk  01 to 2 servings/d  0>=2 servings/d  Consumption  0 < 1 serving/mo  01 to 4 servings/mo  02 to 6 servings/wk  01 to 2 servings/d  0>=2 servings/d  **Women**  Consumption  0 < 1 serving/mo  01 to 4 servings/mo  02 to 6 servings/wk  01 to 2 servings/d  0>=2 servings/d  Consumption  0 < 1 serving/mo  01 to 4 servings/mo  02 to 6 servings/wk  01 to 2 servings/d  0>=2 servings/d  Consumption  0 < 1 serving/mo  01 to 4 servings/mo  02 to 6 servings/wk  01 to 2 servings/d  0>=2 servings/d | **All-cause mortality**  1.00 (reference)  0.95 (0.89-1.00)  1.00 (0.95-1.04)  0.98 (0.91-1.06)  0.99 (0.91-1.07)  **CVD mortality**  1.00 (reference)  0.97 (0.87-1.08)  0.99 (0.91-1.07)  1.11 (0.98-1.26)  1.06 (0.90-1.23)  **Cancer mortality**  1.00 (reference)  1.01 (0.92-1.12)  1.03 (0.95-1.11)  0.99 (0.87-1.13)  0.95 (0.82-1.11)  **All-cause mortality**  1.00 (reference)  0.96 (0.93-1.00)  0.94 (0.91-0.98)  0.97 (0.93-1.02)  1.10 (1.04-1.16)  **CVD mortality**  1.00 (reference)  0.90 (0.82-0.99)  0.89 (0.82-0.98)  0.95 (0.84-1.07)  1.15 (1.01-1.31)  **Cancer mortality**  1.00 (reference)  1.00 (0.94-1.07)  0.95 (0.90-1.01)  0.98 (0.91-1.06)  1.05 (0.96-1.15) | Age, smoking, alcohol intake, postmenopausal hormone use (NHS), physical activity, family history of diabetes, family history of myocardial infarction, family history of cancer, multivitamin use, ethnicity, and aspirin use, baseline history of hypertension and hypercholesterolemia; intake of whole grains, fruit, vegetables, and red and processed meat; total energy, body mass index, and sugar-sweetened beverages consumption. |
| Mossavar-Rahmani et al., 2019^c^ | The Women’s Health Initiative Observational Study | United States | Women  71,926  Men: NA | 50-79 | Mean:11.9 | Questionnaire | All-cause mortality  12,978 | Medical records and National Death Index | Cox proportional hazards regression model | Mean: 0.32 | **White**  Consumption  0 to 1 serving/wk  1 to 4 servings/wk  5 to 7 servings/wk  >=2 servings/d  **Black**  Consumption  0 to 1 serving/wk  1 to 4 servings/wk  5 to 7 servings/wk  >=2 servings/d  **Other**  Consumption  0 to 1 serving/wk  1 to 4 servings/wk  5 to 7 servings/wk  >=2 servings/d | **All-cause mortality**  1.00 (Reference)  0.97 (0.92–1.03)  1.04 (0.97–1.12)  1.22 (1.10–1.36)  1.00 (Reference)  1.07 (0.82–1.39)  1.06 (0.75–1.50)  0.74 (0.41–1.34)  1.00 (Reference)  0.96 (0.73–1.27)  0.99 (0.67–1.47)  1.03 (0.54–1.96) | Age, race, education, health risk variables (diabetes mellitus, CVD, high cholesterol requiring pills, hypertension, BMI), and behaviors (smoking, alcohol, MET, HEI). |
| Vyas et al., 2015^c^ | The Women’s Health Initiative Observational Study | United States | Women  59,614  Men: NA | Mean  62.8 years | Mean:8.7 | FFQ | CVD mortality  942 | Medical records and National Death Index | Cox proportional hazards regression model | Mean: 0.32 | Consumption  0-3 drinks/wk  1-4 drinks/wk  5-7 drinks/wk  >=2 drinks/d  Consumption  0-3 drinks/wk  1-4 drinks/wk  5-7 drinks/wk  >=2 drinks/d | **All-cause mortality**  1.00 (Reference)  1.00 (0.90–1.10)  1.10 (0.90–1.20)  1.30 (1.04–1.50)  **CVD mortality**  1.00 (Reference)  0.90 (0.70–1.20)  0.90 (0.70–1.30)  1.50 (1.03–2.30) | Age, race, education and income, smoking status, BMI, and a history of diabetes, hypertension and hyperlipidemia, alcohol intake, log calibrated energy intake, physical activity, sugar-sweetened beverage intake, salt intake, and hormone therapy. Adjusted cardiac event models were stratified on hypertension, and high cholesterol and adjusted mortality models were stratified on hypertension and the history of diabetes because these variables did not meet the proportional hazards assumption. |
| Paganini-Hill et al., 2007 | The Leisure World Cohort Study | United States | 13,624  Men:4,980  Women:8,644 | Median  74 years | Mean (total participants): 13.2  Men  11.2 ± 6.8  Women  14.3 ± 6.8 | Questionnaire. | All-cause mortality  11,386 | Death indexes and ascertainment of death certificates | Cox proportional hazards regression model | Mean: 0.02 | **Cola**  Consumption  0 can/wk  <=1 can/wk  >1 cans/wk  **Others**  Consumption  0 can/wk  <=1 can/wk  >1 cans/wk | **All-cause mortality**  1.00 (reference)  0.98 (0.93-1.03)  1.18 (1.07-1.30)  1.00 (reference)  0.96 (0.92-1.02)  1.07 (0.97-1.19) | Age, sex, smoking, exercise, body mass index, alcohol intake and histories of hypertension, angina, heart attack, stroke, diabetes, rheumatoid arthritis, and cancer. |

^a^The study by Liu et al. in the UK Biobank was included in the meta-analysis for CVD and cancer mortality but not all-cause mortality, while the study by Anderson et al., also from the UK Biobank, including more participants and examining general artificial sweetened beverages and all-cause mortality but not cause-specific mortality was included in the meta-analysis for all-cause mortality. ^b^ The study by Keller et al did not report the mean level of ASB intake and the mean level also could not be calculated per the information provided in the manuscript. ^c^The study by Vyas et al. in The Women’s Health Initiative Observational Study was included in the meta-analysis for CVD mortality, while the Study by Mossavar-Rahmani et al. also from The Women’s Health Initiative Observational Study including more participants but only examining the associations with all-cause mortality was included in the meta-analysis for all-cause mortality.

# Supplementary Table 3: Inclusion of primary and secondary confounders in adjusted models of included studies

|  | Naomi et al., 2023 | McCullough et al., 2022 | Liu et al., 2022 | Zhang et al., 2021 | Keller et al., 2020 | Anderson et al., 2020 | Mullee et al., 2019 | Malik et al., 2019 | Mossavar-Rahmani et al., 2019 | Vyas et al., 2015 | Paganini-Hill et al., 2007 |
| --- | --- | --- | --- | --- | --- | --- | --- | --- | --- | --- | --- |
| **Primary confounders** | | | | | | | | | | | |
| Age | X | X | X | X | X | X | X | X | X | X | X |
| Sex^a^ | X | X | X | X | X | X | X | X | X | X | X |
| Weight^b^ | X | NA | X | X | X | X | X | X | X | X | X |
| Smoking status | X | X | X | X | X | X | X | X | X | X | X |
| Total energy intake | X | NA | X | X | X | X | X | X | NA | X | NA |
| Alcohol intake | X | X | X | X | X | X | X | X | X | X | X |
| Physical activity | X | NA | X | X | X | X | X | X | X | X | X |
| **Secondary confounders** | | | | | | | | | | | |
| Diet quality score, specific food groups, such as fruits, vegetables, or red meat, or other dietary factors | X | X | X | X | X | X | X | X | X | NA | NA |

^a^ The study by Malik et al., conducted the analysis in men and women, separately. The studies by Mossavar-Rahmani et al., and Vyas et al., conducted the analysis only in women.

^b^ Included BMI, change in weight during follow-up, and body weight.

#

# Supplementary Table 4: Newcastle-Ottawa scale score for each included study

| **Author, year** | **Selection** | | | | **Comparability** | | **Outcome** | | | **Total** |
| --- | --- | --- | --- | --- | --- | --- | --- | --- | --- | --- |
|  | **Representativeness of the exposed cohort^a^** | **Selection of the non-exposed cohort^b^** | **Ascertainment of exposure^c^** | **Outcome of interest present at start of the study^d^** | **Control for primary confounders^e^** | **Control for secondary confounders^f^** | **Assessment of outcome^g^** | **Duration of follow-up^h^** | **Adequacy of follow-up^i^** |  |
| **Unclear risk of bias** | | | | | | | | | | |
| Naomi et al., 2023 | N | Y | BO | Y | Y | Y | Y | Y | Y | 7 |
| McCullough et al., 2022 | N | Y | BO | Y | N | Y | Y | Y | Y | 6 |
| Liu et al., 2022 | N | Y | BF | Y | Y | Y | Y | N | Y | 7 |
| Zhang et al., 2021 | N | Y | BF | N | Y | Y | Y | Y | Y | 7 |
| Keller et al., 2020 | N | Y | BO | Y | Y | Y | Y | N | Y | 6 |
| Anderson et al., 2020 | N | Y | BF | Y | Y | Y | Y | N | Y | 7 |
| Mullee et al., 2019 | N | Y | BO | Y | Y | Y | Y | Y | Y | 7 |
| Malik et al., 2019 | N | Y | BF | Y | Y | Y | Y | Y | Y | 8 |
| Mossavar-Rahmani et al., 2019 | N | Y | BF | Y | N | Y | Y | Y | Y | 7 |
| Vyas et al., 2015 | N | Y | BF | Y | Y | Y | Y | N | Y | 7 |
| Paganini-Hill et al., 2007 | Y | Y | BO | Y | N | N | Y | Y | Y | 6 |

^a^ Representativeness of the exposed cohort: 1 point awarded if community-based population.

^b^ Selection of the non-exposed cohort: 1 point awarded if drawn form the same community as the exposed cohort.

^c^ Ascertainment of exposure: 1 point awarded if diet assessed at baseline and at least one time during follow-up, BO: dietary information collected at baseline visit only; BF: dietary information collected at both baseline and follow-up visit.

^d^ Outcome of interest not present at start of the study: 1 point awarded if individuals with prevalent diabetes, CVD, and cancers at baseline were excluded.

^e^ Control for primary confounders: 1 point awarded if adjustment for age, sex, body mass index (BMI), smoking status, physical activity, alcohol intake, physical activity, and total energy intake.

^f^ Control for secondary confounders: 1 point awarded if adjustment for diet quality (e.g., alternative healthy eating index), or intakes of ≥1 additional food associated with other major foods (e.g., meat, tea, coffee, whole grains, refined grains, fruits, and vegetables).

^g^ Assessment of outcome: 1 point awarded if self-reported cases were confirmed using additional measures (e.g. death certificate, medical records).

^h^ Duration of follow-up; 1 point awarded if follow-up ≥ 10 years.

^i^ Adequacy of follow-up: 1 point awarded if loss to follow-up <20%.

# Supplementary Table 5: Pre-specified subgroup meta-analyses of the association between artificially sweetened beverage consumption and risk of all-cause and cause-specific mortality, using random-effects models.

| **Stratification** | **Categories** | **Risk estimates, n** | **Pooled relative risk**  **(95% CI)** | **I^2^ (%)** | **P-value for interaction^a^** |
| --- | --- | --- | --- | --- | --- |
| **All-cause mortality** | | | | | |
| Age | <60 years | 8 | 1.13 (1.04, 1.23) | 75.0 | 0.93 |
|  | ≥60 years | 4 | 1.16 (1.06, 1.26) | 14.0 |  |
| Number of participants | <100,000 | 5 | 1.07 (0.93, 1.23) | 61.0 | 0.37 |
|  | ≥100,000 | 7 | 1.15 (1.06, 1.25) | 73.1 |  |
| Region | USA | 7 | 1.07 (0.99, 1.16) | 64.0 | 0.05 |
|  | Europe | 5 | 1.23 (1.16, 1.31) | 1.5 |  |
| Sex | Both | 5 | 1.12 (0.99, 1.27) | 62.0 | 0.93 |
|  | Men | 2 | 1.11 (0.88, 1.41) | 91.1 |  |
|  | Women | 5 | 1.16 (1.07, 1.26) | 51.6 |  |
| Follow-up duration | <15 years | 8 | 1.13 (1.02, 1.24) | 51.6 | 0.89 |
|  | ≥15 years | 4 | 1.14 (1.03, 1.26) | 82.9 |  |
| Number of cases | <10,000 | 8 | 1.15 (1.03, 1.28) | 66.4 | 0.65 |
|  | ≥10,000 | 4 | 1.11 (1.02, 1.20) | 76.4 |  |
| Adjusted for total energy intake | No | 4 | 1.16 (1.06, 1.26) | 14.0 | 0.94 |
|  | Yes | 8 | 1.13 (1.04, 1.23) | 75.0 |  |
| Dietary assessment method | 24-hours diet recalls | 4 | 1.06 (0.94, 1.20) | 78.5 | 0.09 |
|  | FFQ | 8 | 1.19 (1.14, 1.25) | 0.0 |  |
| Mean intake of ASB | <0.3 servings/day | 6 | 1.20 (1.13, 1.28) | 18.5 | 0.06 |
|  | ≥0.3 servings/day | 6 | 1.05 (0.95, 1.16) | 68.4 |  |
| **CVD mortality^b^** | | | | | |
| Number of participants | <100,000 | 2 | 1.30 (0.97, 1.75) | 0.7 | 0.87 |
|  | ≥100,000 | 5 | 1.25 (1.07, 1.47) | 64.5 |  |
| Region | USA | 4 | 1.13 (1.03, 1.24) | 0.0 | 0.03 |
|  | Europe | 3 | 1.48 (1.27, 1.73) | 0.0 |  |
| Sex | Both | 2 | 1.10 (0.77, 1.56) | 0.0 | 0.74 |
|  | Men | 2 | 1.26 (0.88, 1.81) | 85.9 |  |
|  | Women | 3 | 1.32 (1.07, 1.63) | 58.4 |  |
| Follow-up duration | <15 years | 3 | 1.26 (0.96, 1.64) | 0.0 | 0.92 |
|  | ≥15 years | 4 | 1.27 (1.07, 1.51) | 73.0 |  |
| Number of cases | <1,000 | 3 | 1.26 (0.96, 1.64) | 0.0 | 0.92 |
|  | ≥1,000 | 4 | 1.27 (1.07, 1.51) | 73.0 |  |
| Dietary assessment method | 24-hours diet recalls | 4 | 1.11 (1.01, 1.22) | 0.0 | 0.02 |
|  | FFQ | 3 | 1.51 (1.31, 1.75) | 0.0 |  |
| Mean intake of ASB | <0.3 servings/day | 3 | 1.48 (1.27, 1.73) | 0.0 | 0.03 |
|  | ≥0.3 servings/day | 4 | 1.13 (1.03, 1.24) | 0.0 |  |
| **Cancer mortality^c^** | | | | | |
| Region | USA | 5 | 0.99 (0.96, 1.02) | 24.3 | 0.23 |
|  | Europe | 3 | 1.07 (0.96, 1.20) | 0.0 |  |
| Sex | Both | 2 | 0.74 (0.53, 1.04) | 0.0 | 0.31 |
|  | Men | 3 | 1.00 (0.94, 1.06) | 21.3 |  |
|  | Women | 3 | 1.00 (0.95, 1.05) | 28.6 |  |
| Follow-up duration | <15 years | 2 | 0.74 (0.53, 1.04) | 0.0 | 0.15 |
|  | ≥15 years | 6 | 0.99 (0.97, 1.02) | 6.8 |  |
| Number of cases | <1,000 | 2 | 0.74 (0.53, 1.04) | 0.0 | 0.15 |
|  | ≥1,000 | 6 | 0.99 (0.97, 1.02) | 6.8 |  |
| Adjusted for total energy intake | No | 2 | 0.98 (0.96, 1.01) | 0.0 | 0.27 |
|  | Yes | 6 | 1.02 (0.94, 1.11) | 28.2 |  |
| Dietary assessment method | 24-hours diet recalls | 2 | 0.74 (0.53, 1.04) | 0.0 | 0.15 |
|  | FFQ | 6 | 0.99 (0.97, 1.02) | 6.8 |  |
| Mean intake of ASB | <0.3 servings/day | 5 | 0.99 (0.97, 1.01) | 0.0 | 0.63 |
|  | ≥0.3 servings/day | 3 | 0.97 (0.83, 1.13) | 56.8 |  |

^a^ P-value for interaction was calculated with meta-regression; p < 0.05 indicated that this difference was statistically significant

^b^ All studies included in the meta-analysis for CVD, and cancer mortality had a mean age of 60 years and below, where we thus did not conduct the subgroups analyses by age.

^c^ Almost all studies included in the meta-analysis for cancer mortality had more than 100,000 participants, where we thus did not conduct the subgroups analyses by numbers of participants.

Supplementary Table 6: Assessment of the quality of evidence on the relationship between artificially sweetened beverage consumption and all-cause and cause-specific mortality using the NutriGrade scoring system.**^a^**

| **CRITERIA** | **Max score** | **Score for ASB and all-cause mortality** | **Score for ASB and CVD mortality** | **Score for ASB and cancer mortality** |
| --- | --- | --- | --- | --- |
| Risk of bias/study quality/study limitations | 2 | 1 | 2 | 1 |
| Precision | 1 | 1 | 1 | 0 |
| Heterogeneity | 1 | 0.5 | 0 | 0 |
| Directness | 1 | 1 | 1 | 1 |
| Publication bias | 1 | 0.5 | 0.5 | 0.5 |
| Funding bias | 1 | 1 | 1 | 1 |
| Effect size | 2 | 0 | 1 | 0 |
| Dose-response | 1 | 1 | 1 | 0 |
| **Overall score** | **10** | 6.0 | 7.5 | 3.5 |

^a^ Evidence grading according to overall score: very-low meta-evidence, 0-3.99; low meta-evidence, 4-5.99; moderate meta-evidence, 6-7.99; high meta-evidence: 8+. The overall score was obtained by summing scores from each component. Maximum possible score for risk of bias/study quality/study limitations, precision, heterogeneity, directness, publication bias, funding bias, effect size, and dose response was 2, 1, 1, 1, 1, 1, 2, and 1, respectively.

# Supplementary Figure 1: Funnel plot for assessment of publication bias for association between artificially sweetened beverage consumption and risk of all-cause and cause-specific mortality.

A: all-cause mortality; B: CVD mortality; C: cancer mortality

P-value for Egger’s test = 0.97; p-value for Begg’s test = 0.84

P-value for Egger’s test = 0.47; ­­p-value for Begg’s test = 1.00

P-value for Egger’s test = 0.98; p-value for Begg’s test = 0.90


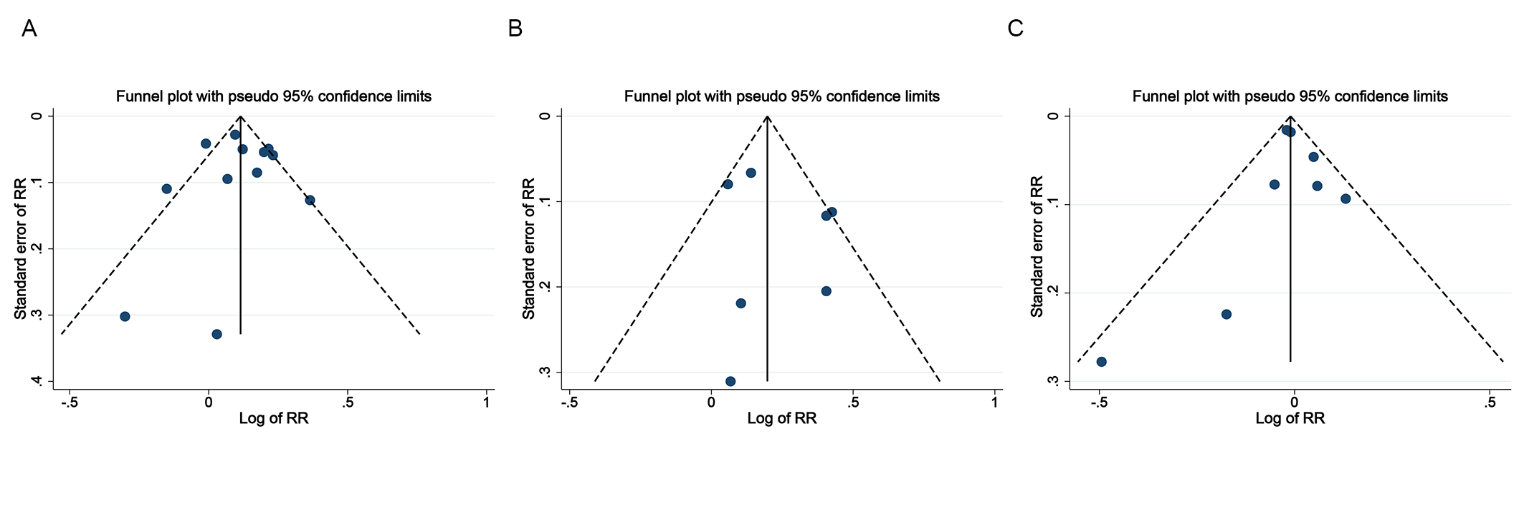


# Supplementary Figure 2: Forest plot of influence analysis for the association between artificially sweetened beverage consumption and risk of all-cause and cause-specific mortality.

A: all-cause mortality; B: CVD mortality; C: cancer mortality


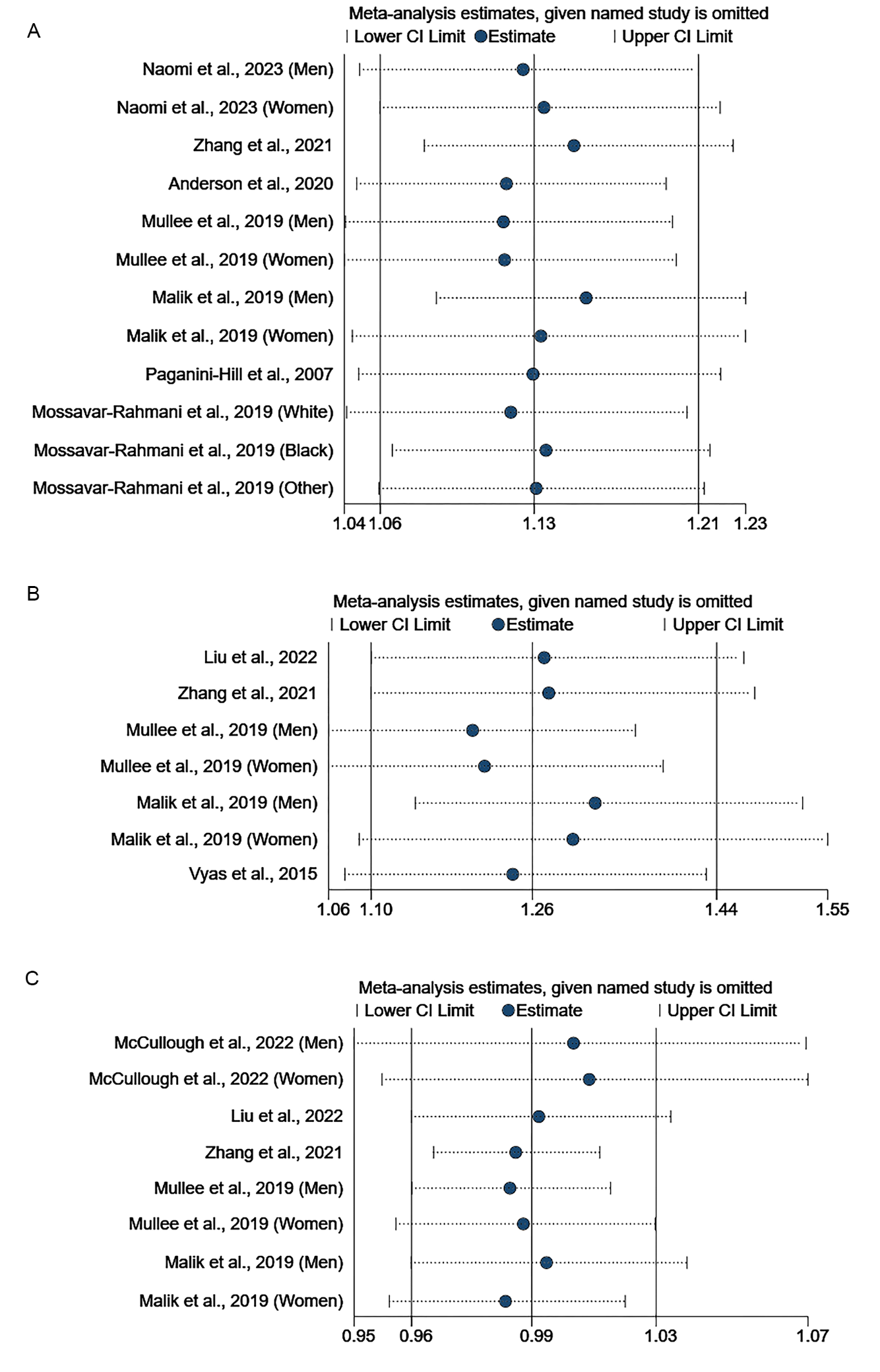

Supplement: Supplementary file 1 — Supplementary Material 1 [file 12937_2024_985_MOESM1_ESM.docx]
